# Supplementary material for: Investigating the influence of physiologically relevant hydrostatic pressure on CHO cell batch culture
Source: Sci Rep. 2021 Jan 8;11:162. doi: 10.1038/s41598-020-80576-8 (PMC7794228; doi:10.1038/s41598-020-80576-8)
Supplement: Supplementary file 1 — Supplementary Information 1. [file 41598_2020_80576_MOESM1_ESM.zip › S1/IgG/Feb14 0 30 60 90/60mmHg Feb3 2.pdf]

Data File C:\Users\P...emStation\1\Data\TK_CHO_IgG 2019-02-14 10-52-34\036-10-ML 60 Feb3.D
Sample Name: ML 60 Feb3

===

Acq. Operator : SYSTEM   Seq. Line : 36
Sample Operator : SYSTEM
Acq. Instrument : HPLC-0039   Location : 10
Injection Date : 2/14/2019 1:25:10 PM   Inj : 1
Inj Volume : 100.000 µl
Sequence File : C:\Users\Public\Documents\ChemStation\1\Data\TK_CHO_IgG 2019-02-14 10-52-34 \TK_CHO_IgG.S
Method : C:\Users\Public\Documents\ChemStation\1\Data\TK_CHO_IgG 2019-02-14 10-52-34 \CHO_IgG_LC.M (Sequence Method)
Last changed : 2/8/2019 3:17:23 PM by SYSTEM

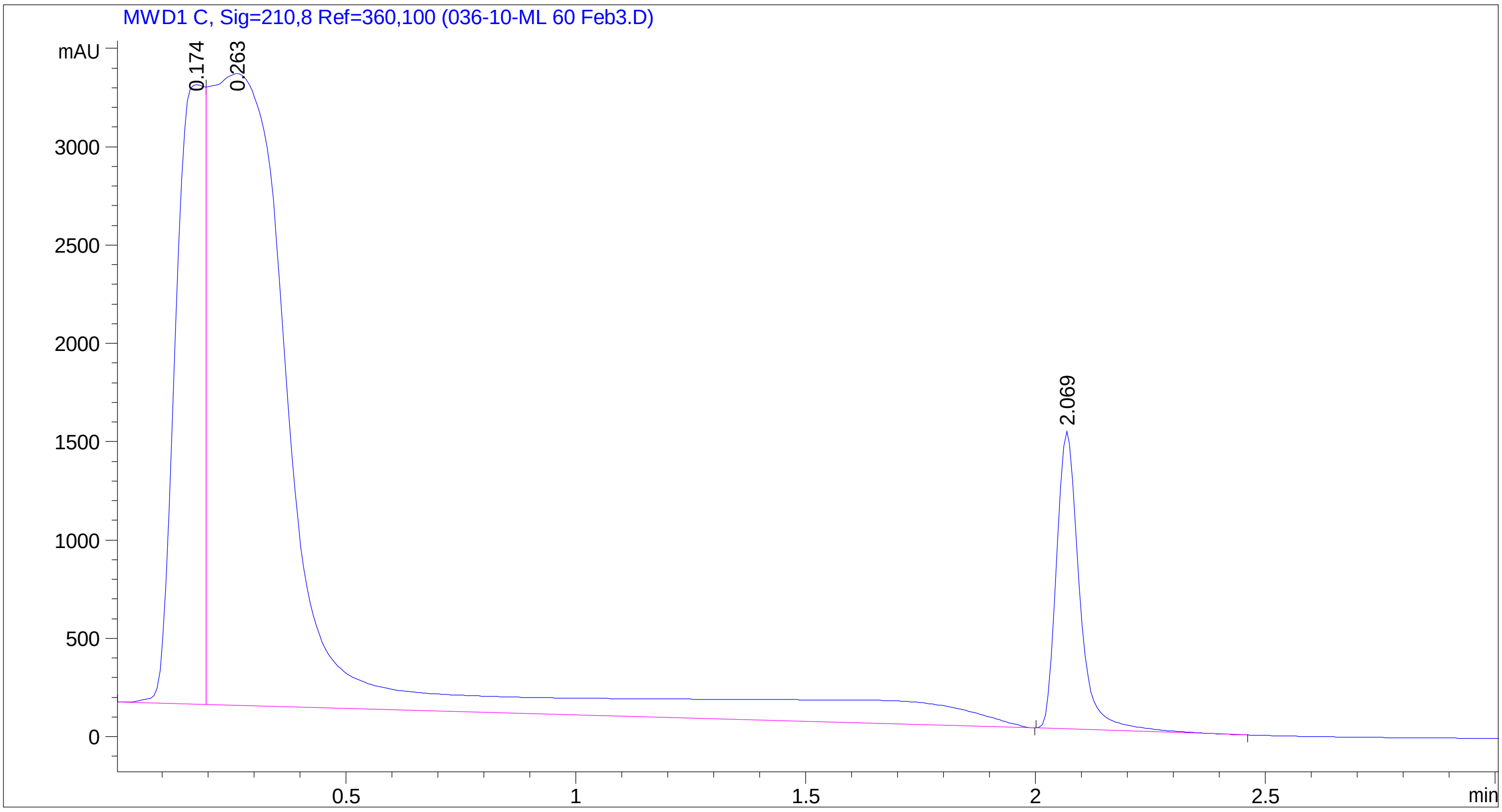

===

Area Percent Report

===

Sorted By : Signal
Multiplier : 1.0000
Dilution : 1.0000
Use Multiplier & Dilution Factor with ISTDs

Signal 1: MWD1 C, Sig=210,8 Ref=360,100

Peak #	RetTime [min]	Type	Width [min]	Area [mAU*s]	Height [mAU]	Area %
1	0.174	BV	0.0684	1.36131e4	3151.19751	21.7053
2	0.263	VB	0.1990	4.37907e4	3213.95361	69.8216
3	2.069	BBA	0.0549	5314.13525	1514.04395	8.4731
Totals :				6.27180e4	7879.19507	

===

*** End of Report ***
